# Danger-Associated Peptide Regulates Root Growth by Promoting Protons Extrusion in an AHA2-Dependent Manner in *Arabidopsis*

**DOI:** 10.3390/ijms21217963

**Published:** 2020-10-27

**Authors:** Nuo Shen, Yanping Jing, Guoqing Tu, Aigen Fu, Wenzhi Lan

**Affiliations:** 1 State Key Laboratory for Pharmaceutical Biotechnology, College of Life Sciences, Nanjing University, Nanjing 210093, China; sn19921112520@163.com (N.S.); 18205098961@163.com (G.T.); 2 College of Life Sciences, Northwest University, Xi’an 710069, China; jingyanping1989@163.com

**Keywords:** *Arabidopsis*, DAMPs, PM H^+^-ATPase, root growth

## Abstract

Plant elicitor peptides (Peps) are damage/danger-associated molecular patterns (DAMPs) that are derived from precursor proteins PROPEPs and perceived by a pair of leucine-rich repeat receptor-like kinases (LRR-RLKs), PEPR1 and PEPR2, to enhance innate immunity and to inhibit root growth in *Arabidopsis thaliana*. In this study, we show that *Arabidopsis* Pep1 inhibits the root growth by interfering with pH signaling, as acidic condition increased, but neutral and alkaline conditions decreased the Pep1 effect on inhibiting the root growth. The perception of Pep1 to PEPRs activated the plasma membrane-localized H+-ATPases (PM H+-ATPases) —the pump proton in plant cell—to extrude the protons into apoplast, and induced an overly acidic environment in apoplastic space, which further promoted the cell swelling in root apex and inhibited root growth. Furthermore, we revealed that pump proton AUTOINHIBITED H^+^-ATPase 2 (AHA2) physically interacted with PEPR2 and served downstream of the Pep1-PEPRs signaling pathway to regulate Pep1-induced protons extrusion and root growth inhibition. In conclusion, this study demonstrates a previously unrecognized signaling crosstalk between Pep1 and pH signaling to regulate root growth.

## 1. Introduction

Under natural conditions, plants’ growth is threatened by kinds of pathogenic bacteria. To protect against pathogens threat, plants have evolved specialized plasma membrane-localized pattern recognition receptors (PRRs) to recognize the pathogen-associated molecular patterns (PAMPs) from pathogens and induce pattern-triggered immunity defense processes [1]. In *Arabidopsis thaliana*, the leucine rich repeat receptor-like kinase (LRR-RLK) FLS2 (flagellin-sensitive 2) recognizes the conserved 22-amino-acid epitope of bacterial flagellin (flg22) to trigger the immune response [2,3]. In addition to PAMPs, plants also release specialized molecules known as danger- or damage-associated molecular patterns (DAMPs), such as cell wall fragments or plant peptides, that could also activate pattern-triggered immunity [4,5]. In *Arabidopsis*, a well-recognized DAMP is a family of plant elicitor peptides (Peps) that are derived from C-terminal regions of their precursor proteins, PROPEPs, and leading the immune responses [6]. The processing of PROPEPs into Peps are mediated by the Ca^2+^-dependent type-II metacaspases (MCs) [7,8]. There are eight Peps (Pep1–Pep8) in *Arabidopsis* that are perceived by two closely related receptor kinases, PEPR1 and PEPR2 [6]. Both PROPEPs and PEPRs are transcriptional-activated by wounding, pathogens infection, or the defense hormone, including salicylate acid (SA) and jasmonic acid (JA) [9,10,11,12]. Among the two receptors, PEPR1 is documented as the predominant receptor for eight Peps in regulating the leaf immunity [6,13]. However, PEPR2 primarily detects Pep1 and Pep2 ligands and functions in root to regulate the root immunity and growth [14,15,16].

The underground roots are surrounded by large rhizosphere pathogenic communities. Timely sensing and resisting rhizosphere pathogenic microorganisms are a prerequisite for plants to survive, however, due to the low accessibility of both the pathogens and the host organs underground, the root immunity is poorly understood [17,18]. The application of PAMPs (such as flg22 and efl18) or DAMPs (such as Peps) could activate root immune response (including elevated cytosolic Ca2+ levels, reactive oxygen species (ROS) bursts, callose deposition, and defense-related gene expression, etc.) and induce the root growth inhibition [14,15,16,19]. Our previous results indicated that the Pep1-PEPRs system triggered a strong root growth inhibition by intersecting with auxin and ROS signaling pathways, both of which were activated by Pep1 treatment that in turn inhibits root growth [15,16].

PM H^+^-ATPases function vitally to regulate plant cell growth, they facilitate an acidic cell wall environment by extrusion of protons to apoplast to promote cell wall expansion [20]. Furthermore, they also provide an energy source for nutrients’ transportation by generating an electrochemical gradient across the plasma membrane [21,22]. PM H^+^-ATPases are encoded by a large gene family, known as *AUTOINHIBITED H^+^-ATPASEs* (*AHA*s), and there are 11 members (*AHA1–11*) in *Arabidopsis thaliana* [23]. *AHA1* and *AHA2′s* functions are more important than other members in roots to maintain ion homeostasis and pH balance in plant cells [24]. Both of the genes express in root to regulate root growth: *AHA1* displays a broad expression domain across the root meristem zone, while *AHA2* mainly locates at the transition zone and elongation zone [20,25]. *AHA7* also expresses in roots, but it has been shown to regulate the root hair growth [26]. The mutants obtained by knocking out these three genes help to assay the PM H^+^-ATPase function in root growth and nutrient uptake. In addition, PM H^+^-ATPases have been reported to regulate the plant immune responses [27,28]. For example, AHA1 and AHA2 cooperated with plant RIN4 protein to regulate the stomatal immunity during pathogen invasion [27]. Bacterial flg22 and fungal chitin could inhibit the PM H^+^-ATPases activity to alkalize the apoplast [27,29].

In the process of revealing the role of Pep-PEPR in plants, we found that binding of Pep1 to PEPR2 induced a strong root growth inhibition, accompanied by swelling epidermal and cortical cells in root transition zone (TZ) [15]. In this study, we revealed that Pep1 treatment inhibited the root growth by activating the PM H^+^-ATPase to extrude the protons into apoplast, which induced an overly acidic environment in apoplast to inhibit root growth. We further revealed that AHA2 was required to regulate the Pep1-induced protons extrusion and root growth inhibition. Finally, we found that AHA2 interacted with PEPR2 both in vitro and in vivo. This study thus demonstrates a previously unrecognized signaling crosstalk between Pep-PEPR and AHA2 in root growth.

## 2. Results

### 2.1. Pep1 Inhibits the Root Growth Dependent on pH Changes

**Figure 1:**
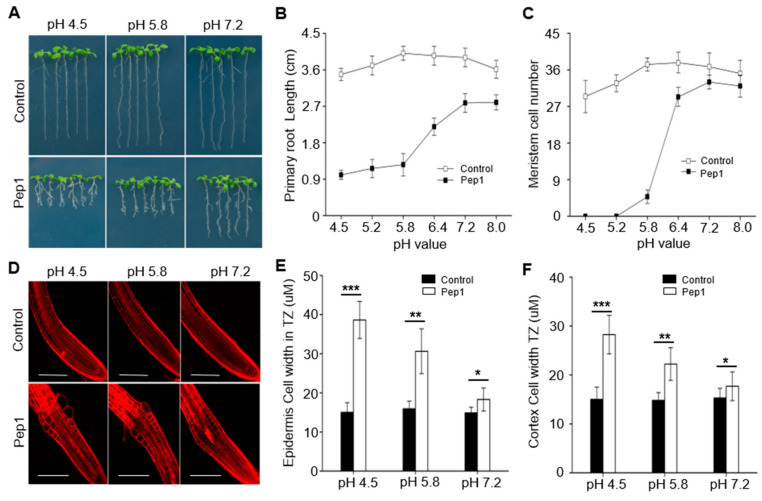
Pep1 inhibits the root growth dependent on the pH changes. The growth phenotype (**A**) and the length of primary roots (**B**) of the three-day-old wild-type (WT) plants were transplanted on half-strength Murashige and Skoog (MS) agar medium in different pH value supplemented with or without (control) 100 nM Pep1 for 6 days. (**C**) Statistics of the number of meristematic cells in root cortex, as in (**B**). Data in (**B**) and (**C**) are means ± SD from three independent experiments (*n* = 15, n represents the number of samples). (**D**) Pep1-induced cell swelling in root transition zone. Five-day-old WT seedings were transferred onto half-strength MS agar medium in different pH value supplemented with or without (control) 100 nM Pep1 for 12 h. The roots were stained with 5 uM propidium iodide (PI) for 15 s and photographed under a confocal laser-scanning microscope. The experiments were repeated three times with similar results. Bars = 100 um. (**E**) and (**F**) Quantitative analysis of epidermal (**E**) and cortex cell (**F**) width in TZ as in (**D**). Data are means ± SD (*n* = 32 cells from 8 roots per treatment). Asterisks in (**E**) and (**F**) indicate statistically significant differences compared with the untreated control (Tukey’s test; * *p* < 0.05; ** *p* < 0.01; *** *p* < 0.001).

The root growth is affected by pH changes. Our previous results indicated that Pep1 triggered a strong root growth inhibition accompanied by swelling epidermal and cortex cells in root apex and decreasing root meristem size [15]. To investigate whether Pep1 interacts with pH signaling to regulate the root growth, we examined the effects of 100 nM Pep1 in the plant growth medium with different pH (4.5 to 8.0) on the growth of wild-type (WT) seedlings. Pep1 treatment induced a significant root growth inhibition under normal pH (5.8) condition (Figure 1A,B), in agreement with previous results [15]. Interestingly, we found that Pep1’s effect on root growth was dependent on the pH changes, as decreasing of medium pH (4.5–5.8) further aggravated the Pep1-induced root growth inhibition, however, increasing of medium pH into faintly acid (pH 6.4), neutral (pH 7.2), or alkali (pH 8.0) condition significantly alleviated the Pep1 effect on root growth (Figure 1A,B).

Continuous root growth and development are sustained by cell division capacity and differentiation rate in the root meristem zone (MZ) and transition zone (TZ) [30,31]. Our previous results showed that root growth inhibition induced by Pep1 was mainly reflected by decreasing of meristem size and promoting cell expansion in TZ [15]. To evaluate whether pH changes interact with Pep1 signaling to affect the cell division capacity and root structure, we assessed the root meristem size by counting the number of meristematic cortex cells between the quiescent center (QC) and the first elongation cell [32]. We found that pH changes in medium determined the Pep1 effect on changing root meristem size; under low pH (4.5–5.2) condition, the root meristem almost disappeared with Pep1 treatment, however, the high pH (6.4–8.0) condition significantly alleviated the Pep1 effect (Figure 1C). We further analyzed the cell expansion in root apex TZ by using propidium iodide (PI), a dye cannot pass through the PM of living cells and labels the cell walls, to visualize cell structure [33,34]. We stained the wild-type root with 5 uM PI and found that epidermal and cortex cells in TZ swelled after Pep1 treatment, however, the low pH (4.5) aggravated but the high pH (7.2) relieved the Pep1 effect on cell swelling (Figure 1D–F). Taken together, these results suggest that Pep1 signaling interacts with the pH signaling pathway to inhibit the root growth.

### 2.2. Pep1-PEPR Promotes the Acidification of Apoplast in Root Apex

**Figure 2:**
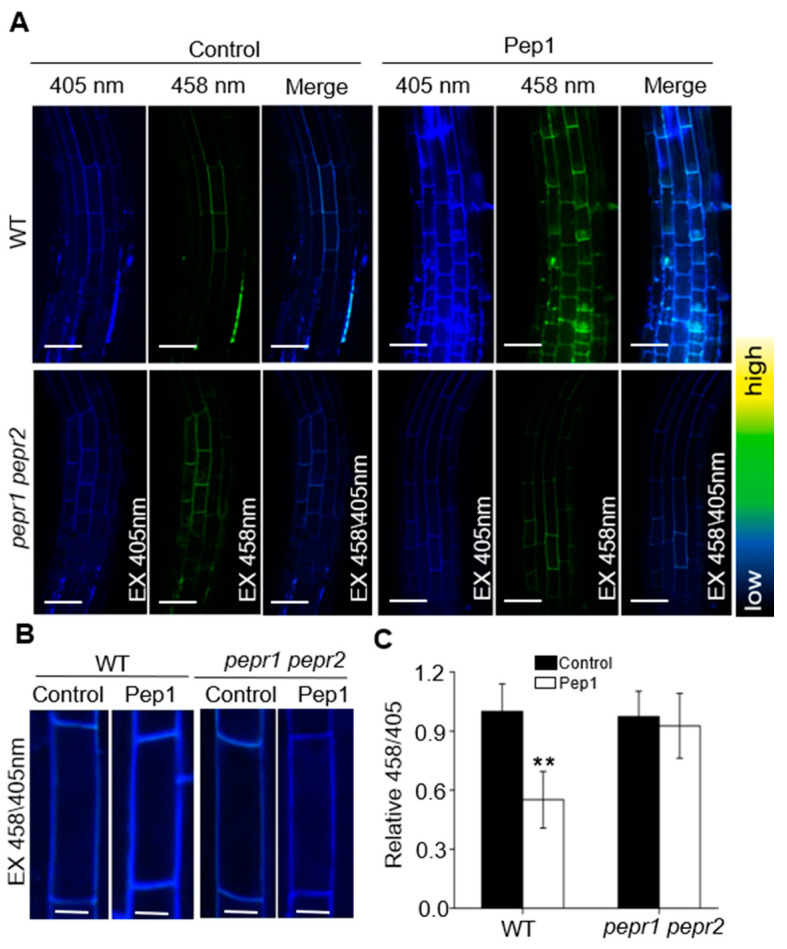
Pep1-PEPR induces the acidification of apoplast in root apex. (**A**) The 8-hydroxypyrene-1,3,6-trisulfonic acid trisodium salt (HPTS) fluorescence signals in 6-day-old wild-type (WT) and *pepr1 pepr2* roots treated with or without (Control) 100 nM Pep1 for 8 h. The root-apex fluorescent signals represented protonated HPTS (EX405) (Excitation 405 nm, emission peak 514 nm), deprotonated HPTS form (EX458) (Excitation 458 nm, emission peak 514 nm), and the merge of EX458/EX405, Bars = 50 μm. Color code (black to yellow) depicts (low to high) 458/405 intensity. (**B**) Representative pictures of the cortex cells in transition zone, Bars = 10 μm. (**C**) The summarized values on fluorescent ratio of deprotonated HPTS to protonated HPTS in the cortex cells of transition zone, as indicated in (B). The fluorescent ratio of the control (WT) was set to 1.0, and the Pep1 treatment ratio was normalized to the control ratio. Data are mean ± SD from three independent experiments (*n* = 16 cells from 8 roots per treatment). Asterisks indicate statistically significant differences compared with Control (Tukey’s test; ** *p* < 0.01).

Cellular growth in plants is constrained by the cell wall, which is the first cellular compartment and occupies most of the apoplastic spaces and provides form and stability for plant cells [35,36]. The acidification of the apoplast promotes the loosening of the cell wall and enables cellular expansion, which is necessary for controlling the TZ position and regulating the root meristem size [20,37]. In this study, we found that Pep1 intersected with pH signaling to promote the cell swelling in TZ and regulate the root meristem size. To investigate whether Pep1 regulates the pH environment in apoplast, we detected the apoplastic pH at a cellular resolution in root apex by using 1 mM 8-hydroxypyrene-1,3,6-trisulfonic acid trisodium salt (HPTS), a suitable fluorescent pH indicator with excitation wavelengths of 405 and 458 nm to visualize the protonated and deprotonated signals, respectively [37]. Under normal growth condition (pH 5.8), the protonated HPTS signals (excitation, 405 nm) was stronger than deprotonated HPTS signals (excitation, 458 nm) in root apex (Figure 2A), in agreement with the previous results [37]. In the presence of 100 nM Pep1, the protonated HPTS signals in wild-type root significantly increased, the 458/405 ratio in root apex TZ was reduced more than 40% compared with untreated control (Figure 2A), suggesting a decrease of apoplastic pH in root apex after Pep1 treatment. However, the protonated HPTS signals and the 458/405 ratios in *pepr1 pepr2* roots were not affected by Pep1 treatment (Figure 2B,C). Taken together, these results suggest that Pep1-PEPR promotes the acidification of the apoplast in root apex.

### 2.3. Pep1 Activates the PM H^+^-ATPase Activity to Regulate Root Growth

**Figure 3:**
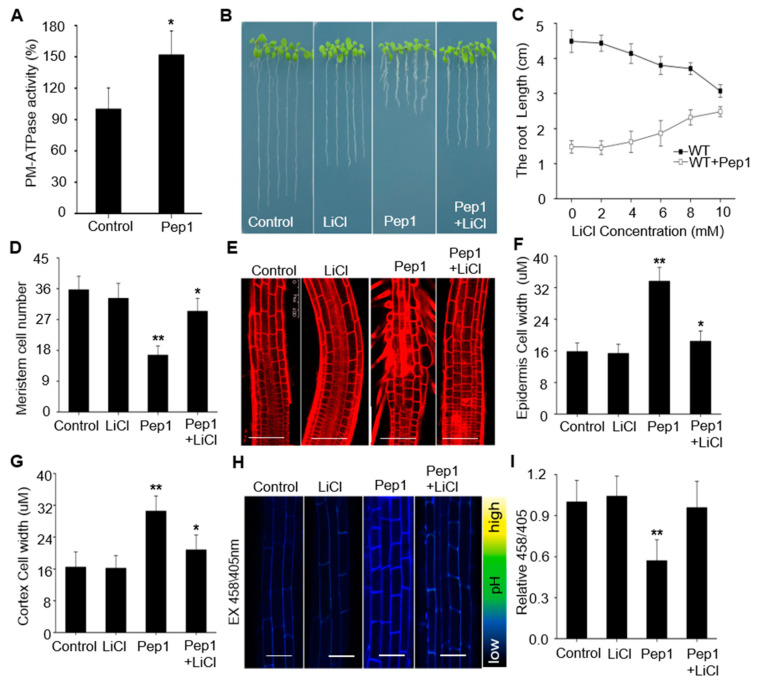
Pep1 activates the plasma membrane-localized H+-ATPases (PM H+-ATPases) to inhibit root growth. (**A**) The PM H^+^-ATPase activity in microsomal membranes isolated from 5-week-old wild-type (WT) roots treated with 100 nM Pep1 for 72 h, the PM H^+^-ATPase activity in control condition was normalized to 1.0. Data are mean ± SD of three independent experiments. The growth phenotype (**B**) and the length of primary root (**C**) of three-day-seedings were transferred on half-strength MS agar medium supplemented with or without (Control) 10 mM LiCl, 100 nM Pep1, or 10 mM LiCl + 100 nM Pep1 for 6 days. (**D**) Statistics of the number of meristematic cells in root cortex as in (**B**). Data in (**B**), (**C**,**D**) are means ± SD from three independent experiments (*n* = 24, n represents the number of samples). (**E**) The root longitudinal structures in WT seedlings. Five-day-old seedings were transferred on half-strength MS agar medium supplemented with or without (Control) 10 mM LiCl, 100 nM Pep1, or 10 mM LiCl + 100 nM Pep1 for 24 h. the roots were stained with 5 uM PI for 15 s and photographed under a confocal laser-scanning microscope. Bars = 100 um. (**F**,**G**) Quantitative analysis of epidermal and cortex cell width in TZ as indicated in (**E**). Data are means ± SD (*n* = 32 cells from 8 roots per treatment). (**H**) HPTS fluorescence signals in 6-day-old WT roots treated with or without (Control) 10 mM LiCl, 100 nM Pep1, or 10 mM LiCl + 100 nM Pep1 for 8 h. The root-apex fluorescent signals represented protonated HPTS (EX405) (Excitation 405 nm, emission peak 514 nm), deprotonated HPTS form (EX458) (Excitation 458 nm, emission peak 514 nm), and the merge of EX458/EX405. Color code (black to yellow) depicts (low to high) 458/405 intensity. Bars = 20 um. (**I**) The summarized values on fluorescent ratio of deprotonated HPTS to protonated HPTS in the cortex cells of transition zone as indicated in (**H**). The fluorescent ratio of the control was set to 1.0, and the treatment ratio was normalized to the control ratio. Data are mean ± SD from three independent experiments (*n* = 16 cells from 8 roots per treatment). Asterisks indicate statistically significant differences compared with Control (Tukey‘s test; * *p* < 0.05, ** *p* < 0.01).

The PM H^+^-ATPases facilitate an acidic cell wall environment by extrusion of protons to apoplast, which is essential for cell wall expansion and cell growth [20]. To explore whether Pep1 affect the PM H^+^-ATPases activity to regulate the root growth, we firstly examined the H^+^-ATPase activity in PM vesicles isolated from wild-type roots. As compared to normal growth condition, the PM H^+^-ATPase activity increased more than 45% under Pep1 treatment (Figure 3A), suggesting that Pep1 treatment increases the PM H^+^-ATPase activity.

To clarify whether Pep1-induced activation of PM H^+^-ATPase attributes to root growth inhibition, we treated the wild-type seedling roots with Pep1 in the presence of various concentrations of Lithium chloride (LiCl), an inhibitor of PM H^+^-ATPase that blocks the protons’ extrusion [38,39]. As shown in Figure 3B–G, addition of LiCl, especially at 10 mM concentration, blocked the Pep1 effects on inhibiting root growth, meristem size decreasing, and cell swelling in TZ. In addition, we confirmed that Li^+^, but not other cations, including K^+^, Na^+^, and Cs^+^, specifically acted in the Pep1 signaling pathway (Supplementary Figure S1). Furthermore, we found that LiCl could block the Pep1-induced apoplast acidification in root apex (Figure 3H,I). Taken together, these results support the hypothesis that Pep1 activates the PM H^+^-ATPase to extrude the protons into apoplast and inhibit root growth.

### 2.4. AHA2 Is Required to Regulate the Pep1 Signaling in Root Growth

**Figure 4:**
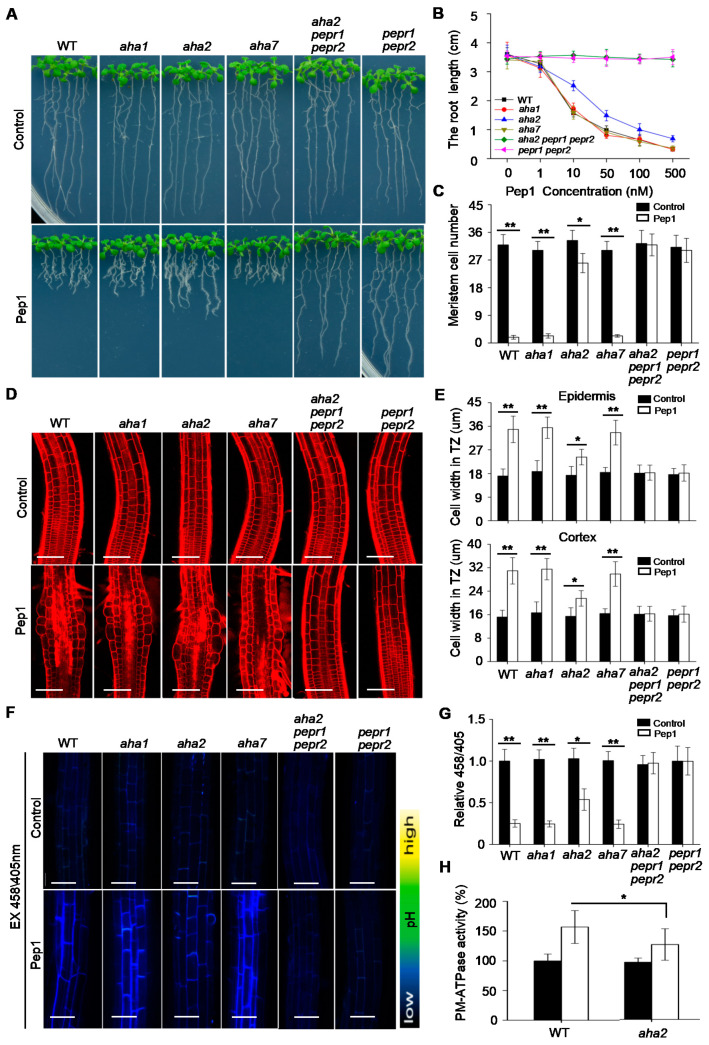
AHA2 is required to regulate the Pep1 signaling in roots. (**A**) The growth phenotype of wild-type (WT), *aha1*, *aha2*, *aha7*, *aha2 pepr1 pepr2,* and *pepr1 pepr2* root. Three-day-old seedings were transferred on half-strength MS agar medium supplemented with or without (Control) 100 nM Pep1 for 6 days. (**B**) The statistical analysis of the primary root length in WT, *aha1*, *aha2*, *aha7*, *aha2 pepr1 pepr2,* and *pepr1 pepr2* plants. The 3-day-old seedlings were transplanted on half-strength MS agar medium supplemented with various concentrations of Pep1 (0–500 nM) for 6 days. Data are means ± SD from three independent experiments, (*n* = 15, *n* represents the number of samples). (**C**) Quantitative analysis of meristematic cortex cells numbers in TZ as indicated in (A). Data are means ± SD from three replicate experiments (*n* = 24). (**D**) Roots’ longitudinal structures in WT, *aha1*, *aha2*, *aha7*, *aha2 pepr1 pepr2,* and *pepr1 pepr2* seedlings. Four-day-old seedings were transferred on half-strength MS agar medium supplemented with or without (Control) 100 nM Pep1 for 24h, the roots were stained with 5 uM PI for 15 s and photographed under a confocal laser-scanning microscope. Bars = 100 um. (**E**) Quantitative analysis of epidermal and cortex cell width in TZ as indicated in (**D**). Data are means ± SD from three independent experiments (*n* = 32 cells from 8 roots per treatment). (**F**) HPTS fluorescence signals in 6-day-old WT, *aha1*, *aha2*, *aha7*, *aha2 pepr1 pepr2* and *pepr1 pepr2* roots treated with or without (Control) 100nM Pep1 for 8h. The root-apex fluorescent signals represented protonated HPTS (EX405) (Excitation 405 nm, emission peak 514 nm), deprotonated HPTS form (EX458) (Excitation 458 nm, emission peak 514 nm), and the merge of EX458/EX405. Color code (black to yellow) depicts (low to high) 458/405 intensity. Bars = 20 um. (**G**) summarized values on fluorescent ratio of deprotonated HPTS to protonated HPTS in the transition zone cortex cells as indicated in (**F**). The fluorescent ratio of the control was set to 1.0, and the Pep1 treatment ratio was normalized to the control ratio in each seedlings. Data are mean ± SD from three independent experiments (*n* = 16 cells from 8 roots per treatment). (**H**) The PM H^+^-ATPase activity in microsomal membranes isolated from 5-week-old wild-type (WT) and *aha2-4* mutant roots treated with 100 nM Pep1 for 72 h, the PM H^+^-ATPase activity in control condition was normalized to 1.0. Data are mean ± SD from three independent experiments (*n* = 3). Asterisks in (**C**), (**E**), (**G**) and (**H**) indicate statistically significant differences (Tukey’s test; * *p* < 0.05, ** *p* < 0.01).

PM H^+^-ATPases are encoded by the *AHAs* (*AHA1* to *AHA11*) gene family in *Arabidopsis* [24]. Among them, *AHA1*, *AHA2,* and *AHA7* have been reported to function in root to regulate the root development [20,25,40]. Our finding of Pep1-induced activation of PM H^+^-ATPases led us to examine the possibility that these three *AHA* (*AHA1, AHA2,* and *AHA7*) genes function in root to regulate Pep1 signaling. To this end, we challenged the *aha1*, *aha2,* and *aha7* single mutant seedlings with Pep1 treatment to analyze the root growth (Supplementary Figure S2). There were no significant differences in root growth between mutants and wild-type seedlings grown under normal condition (Figure 4A,B). When supplemented with various concentrations of Pep1 (ranged from 1 to 500 nM), we found that *aha1* and *aha7* mutant roots displayed similar responses to Pep1 as wild-type plants, however, the roots growth in *aha2* mutant was shown to decrease the Pep1 sensitivity (Figure 4A,B). We further analyzed the root meristem size and found that root meristem in wild type, *aha1,* and *aha7* was almost abolished under 100 nM Pep1 treatment, and the meristem size in *aha2* mutant decreased by 30% (Figure 4C). Moreover, the Pep1-induced cell swelling in TZ was suppressed by disruption of AHA2, but not AHA1 and AHA7 (Figure 4D,E). In addition, we also analyzed the root growth in *aha2 aha7* double mutant, but not *aha1 aha2* mutant for its embryo lethality [24]. We excluded the functional redundancy between AHA2 and AHA7 in regulating the Pep1 signaling, since the double mutant did not further alter the Pep1 effects as compared to *aha2* plants (Supplementary Figure S3). Taken together, these results suggest that AHA2 is required to regulate the Pep1 signaling in root.

To explore whether AHA2 contributes to proton secretion in root apex under Pep1 treatment, we analyzed the apoplastic acidification in wild-type and *aha2* roots. Through HPTS staining, we found that the apoplastic pH in root apex did not display difference between *aha2* and wild-type seedlings (Figure 4F,G). The disruption of AHA2 inhibited the Pep1 effect on promoting apoplastic acidification (Figure 4F,G). In addition, the disruption of the other two AHAs, AHA1 and AHA7, did not alter the Pep1 effect (Figure 4F,G). Furthermore, we assayed the PM H^+^-ATPase activity and found that the loss-of-function of AHA2 did not alter the PM H^+^-ATPase activity in root under control conditions (Figure 4H), consistent with the previous studies [25]. In the presence of 100 nM Pep1, despite that PM H^+^-ATPase activities were increased both in wild-type and *aha2* seedlings, the increasing level in *aha2* was lower than that in wild-type root (Figure 4H). In conclusion, these results suggest that AHA2 plays a vital role to regulate the proton extrusion in response to Pep1 signaling.

Pep1 is perceived by its receptor PEPR1 and PEPR2 to initiate the cell immune and growth signaling [6]. In order to get more information on the genetic relationship between AHA2 and PEPRs, we constructed an *aha2 pepr1 pepr2* triple mutant by crossing the *aha2* with the *pepr1 pepr2* mutant (Supplementary Figure S4). We challenged the triple mutant seedlings with Pep1 treatment and found that primary root length, root meristem size, cell expansion in TZ, and proton secretion in *aha2 pepr1 pepr2* triple mutant were similar to those in *pepr1 pepr2* seedlings, and both of the materials were completely insensitive to Pep1 treatment (Figure 4). These results demonstrated that the AHA2 acts downstream of PEPRs to respond to Pep1 signaling.

### 2.5. PEPR2 Interacts with AHA2 In Vitro and In Vivo

**Figure 5:**
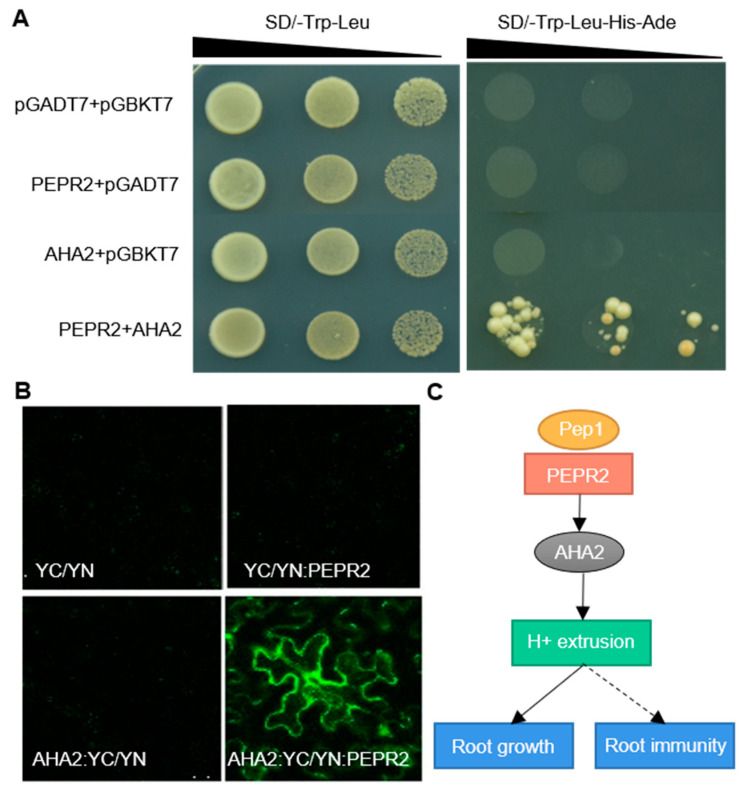
PEPR2 interacts with AHA2. (**A**) Yeast two-hybrid assay of protein interaction between PEPR2 and AHA2. Saturated cultures were spotted onto synthetic dropout (SD)-Trp-Leu and SD-Trp-Leu-His-Ade at different dilutions (10^−1^, 10^−2^, and 10^−3^). The co-transformants of the empty vectors pGADT7 and pGBKT7 were employed as negative controls. (**B**) Bimolecular fluorescence complementation of pSPYCE(M)/pSPYNE(R)173 (YC/YN), pSPYCE(M)/pSPYNE(R)173: PEPR2 (YC/YN:PEPR2), AHA2: pSPYCE(M)/pSPYNE(R)173 (AHA2:YC/YN), and AHA2: pSPYCE(M)/pSPYNE(R)173: PEPR2 (AHA2:YC/ YN:PEPR2) Images were taken using confocal laser scanning microscopy. Bars = 50 μm. (**C**) A proposed model of the Pep1-PEPR2 signaling pathway activates the PM H^+^-ATPase in roots.

Among the two receptors of Pep1, PEPR1 plays a more prominent role than PEPR2 in mediating Pep1-induced defense responses in leaves [13], while PEPR2 is a major player in root to perceive Pep1 signaling (Supplementary Figure S5) [15]. In this study, we proved that AHA2 acted downstream of PEPRs to regulate the Pep1 signaling. To further confirm the connection between AHA2 and PEPR2, we performed a yeast two-hybrid experiment to detect the protein interactions between the two proteins. AHA2 protein contains 948 amino acids that integrate into the membrane to form 10 transmembrane segments and has most of its remaining mass, including the N- and C-terminal domains, exposed on the cytosolic side of the membrane [41]. The C-terminal serves as a kinase domain (KD) to regulate enzymatic activity [41]. We cloned the C-terminal (containing amino acids 836–948) of AHA2 as bait and the C terminus of PEPR2 KD (containing amino acids 760–1072) as prey and found that AHA2 interacted with PEPR2 in vitro (Figure 5A). Furthermore, we verified this interaction in vivo by using the bimolecular fluorescence complementation (BiFC) system. We transformed the pSPYNE(R)173: PEPR2 (YN:PEPR2) and AHA2: pSPYCE(M) (AHA2:YC) plasmid into *Nicotiana benthamiana* leaves and found that PEPR2 could also directly interact with AHA2 (Figure 5B). Taken together, these results demonstrate that PEPR2 interacts with AHA2 both in vitro and in vivo.

## 3. Discussion

The pathogenic bacteria in rhizospheres surround the plant roots and threaten plant health. Plants protect themselves against pathogen threats by perception of PAMPs or DAMPs via the hosts’ plasma membrane-localized pattern recognition receptors (PRRs) to activate the roots immune responses. The activation of immune signals in roots is usually accompanied by root growth inhibition. However, relatively little is known about this growth–immunity trade-off in roots. In this study, we showed that *Arabidopsis* Pep1 suppresses the root growth by intersecting with pH signaling. The perception of Pep1 via PEPRs triggers a strong acidification of apoplast by activating the AHA2, a PM H^+^-ATPase, to promote the protons’ extrusion, which further regulate root growth by altering cell expansion and differentiation. These findings reveal a previously unrecognized signaling pathway by which danger peptides regulate root growth.

Plant cells are surrounded by a rigid cell wall, which needs to undergo loosening to allow cellular expansion [37]. The long-standing acid growth theory shows that PM H^+^-ATPase-mediated proton extrusion decreases the pH in intercellular space, which activates cell wall loosening enzymes and enables cell expansion [37,42]. Upon pathogens infection, the roots activate the immune responses accompanied by stopping the cell growth, and apoplast acidification plays a vital role in mediating root growth inhibition [43]. The plant immunity-related Pep1 treatment could also trigger the root growth inhibition by swelling cells in the root apex [15]. In the current study, we found that Pep1 treatment induced the acidification of apoplastic space in the root apex, which promoted the cell swelling and inhibited root growth (Figure 1 and Figure 2). The pH altering in root apoplast changed the Pep1 effect, as acid increased, but neutral or alkaline condition alleviated the Pep1 effect on root growth inhibition (Figure 1). Pep1 is released form it’s precursor protein PROPEP1 during pathogens’ induction [7,8]. The immunity-related root growth inhibition could be explained by activating the Pep1 signaling to acidify the apoplast in the root apex and inhibit root growth.

PM H^+^-ATPases are primary proton pumps responsible for establishment of proton electrochemical gradient across the plasma membrane [44]. In *Arabidopsis*, PM H^+^-ATPases have been reported to regulate the plant immune responses. AHA1 and AHA2 regulate the leaf immunity by controlling stomatal apertures, and constitutively active AHA prevents stomatal closure in response to flg22 or *P. syringae* effector AvrB treatment, which favors pathogens invasion [27,45]. To this end, the downregulation of PM H^+^-ATPase activity seems to be helpful to improve plant immunity in leaf tissues [46]. However, in this study, we found that Pep1 treatment activated the PM H^+^-ATPase to extrude the proton into apoplast, which promoted the acidification of apoplast in root apex cells (Figure 2 and Figure 3), those changes are different with flg22 or RAPID ALKALINIZATION FACTORs (RALFs) effects on triggering extracellular alkalinization by inhibiting PM H^+^-ATPase activity with transient treatment [47,48]. Pep1 signaling has been termed as an amplifier of flg22 signaling in plant leaves [11,13], but the two show differences in roots to regulate the PM H^+^-ATPase activity. Whether the Pep1-induced activation of PM H^+^-ATPase is required to regulate the root immunity demands a more in-depth examination.

In *Arabidopsis*, the proton pumps AHA1 and AHA2 function vitally in root to regulate the ion homeostasis and cell growth [24]. AHA1 displays a broad expression domain across the root meristem zone, while AHA2 mainly locates at the root transition and elongation zone [20,25]. In the current study, we found that AHA2 is required to regulate the Pep1 signaling in root growth, as disruption of AHA2, but not AHA1 blocked the Pep1 effect in roots (Figure 4). The activity of AHA2 is determined by the Thr881 and Ser899 sites at the C-terminal, and flg22 treatment induces an increase of Ser899 phosphorylation but a decrease of Thr881 phosphorylation in AHA2 protein [41,46]. AHA2 is also termed as a new component in the FLS2 receptor complex [49]. We found that AHA2 acted downstream of PEPRs to respond to the Pep1 signaling (Figure 5). PEPR2 interacted with AHA2 both in vitro and in vivo (Figure 5), and those results provide evidence that AHA2 may acts as a direct substrate of PEPR2, but the potential phosphorylation sites are unknown and need to be further explored. According to our results, we propose a model of the Pep1-PEPRs signaling pathway that regulates root growth by activating the PM H+-ATPase, with the perception of Pep1 to PEPR2 leading to interact with and phosphorylate the proton pump AHA2 to activate the proton extrusion, which acidifies the apoplast space to inhibit the root growth, as shown in Figure 5C.

In conclusion, the results of this study have uncovered a significant role of the Pep1-PEPR system, in which it cooperates with PM H^+^-ATPase to regulate root growth. It will be interesting to further investigate the potential phosphorylation mechanism by which Pep1-PEPR regulates the AHA2 activity and confirm the function of AHA2 in mediating Pep1-induced root immunity. The research will enrich the root immunity theory and increase researcher cognition of how dose plant elicitor peptide regulates the proton pump activity to mediate root immunity and root growth.

## 4. Materials and Methods

### 4.1. Plant Materials and Growth Conditions

*Arabidopsis thaliana* wild-type (WT; ecotype Columbia-0) and the T-DNA insertion mutants *aha1–7* (SALK_065288) and *aha2–4* (SALK_082786) were obtained from the *Arabidopsis* Biological Resource Center (ABRC). The mutant *aha7* [25] and the *pepr1 pepr2* [6] double mutants were described in previous studies. All *Arabidopsis* lines in this study were Columbia (Col-0) ecotype background. *aha2 aha7* and *aha2 pepr1 pepr2* homozygous mutants were obtained by hybridization and identified by polymerase chain reaction (PCR) using the primers listed in Supplementary Table S1.

For on-plate growth assays, seeds were sterilized with 75% (*v/v*) ethanol for 3 min, two times, washed in sterilized water, and sown on half-strength Murashige and Skoog (1/2 MS) medium containing 1% (*w/v*) sucrose (Aldrich-Sigma, St. Louis, MI, USA) and solidified 0.8% (*w/v*) Phytagel (Sigma-Aldrich, St. Louis, MI, USA). In order to break the dormancy of seeds and make seeds germinate easily, the plates were incubated at 4 °C in darkness for 2 days and then were positioned vertically in a growth chamber with a 16 h light/8 h dark regime with white fluorescent tubes (Philips, Amsterdam, Netherlands) at a light intensity of 90 µmol/m^2^/s at 22 °C.

### 4.2. Peptides

Pep1 (ATKVKAKQRGKEKVSSGRPGQHN) obtained from Sangon Biotech (Sangon Biotech, Shanghai, China) was dissolved in water (stock solutions of 1 mM).

### 4.3. Reverse Transcription PCR (RT-PCR) Analysis

Total RNA in leaves and roots were extracted using the TRIzol reagent (Invitrogen, Carlsbad, CA, USA), according to the manufacturer’s protocol. The 2 μg RNA was used to synthesis the cDNA by using Moloney Murine Leukemia Virus (M-MLV) Reverse Transcriptase (Promega, Madison, WI, USA). The resulting cDNA was used for PCR amplification to clone related genes or verify absence of transcript in mutants with the gene-specific primers (Supplementary Table S1) on a T100 thermal cycler (Bio-Rad, Berkeley, CA, USA)). PCR products were separated on a 1% (*w/v*) agarose gel stained with ethidium bromide (Sangon Biotech, Shanghai, China).

### 4.4. Root Structure Analysis

For propidium iodide staining, 6-day-old seedlings were transferred onto half-strength MS agar medium supplemented with 100 nM Pep1 for 24 h. The roots were stained with 5 uM propidium iodide solution for 15 s, rinsed 3 times, and photographed under an confocal microscope (LSM-710, Zeiss, Oberkochen, Germany) with the excitation wavelength of 543 nm. The cell width was measured by using Image J 1.51K Software (National Institutes of Health, Bethesda, MD, USA).

### 4.5. Apoplast Acidification Analysis

To detect apoplast acidification, the HPTS (8-hydroxypyrene-1,3,6-trisulfonic acid trisodium salt) staining method was used to visualize apoplastic pH, as described previously, with modification [37]. Briefly, 7-day-old *Arabidopsis* seedlings were transferred onto half-strength MS agar medium (pH 5.8) without or with 100 nM Pep1 for 8 h, and then incubated in 1 mM HPTS (pH 5.8) for 30 min. After rinsing with H_2_O three times, the roots were observed under a confocal microscope (LSM-710, Zeiss, Oberkochen, Germany). The protonated HPTS form (excitation 405 nm, emission peak 514 nm) and deprotonated HPTS form (excitation 458 nm, emission peak, 514 nm) were detected. Image analysis was performed using the Image J 1.51K Software (National Institutes of Health, Bethesda, MD, USA).

### 4.6. Measurements of PM H^+^-ATPase Activity

Five-week-old *Arabidopsis* plants were transferred onto 1/6 MS solution without sucrose (pH 5.8) supplemented with 0 or 20 nM Pep1. After 5 days of treatment, roots were collected to prepare the plasma membrane vesicles as described previously [50]. We used 10 µM vanadate to evaluate feasibility and found that the vanadate-sensitive ATPase occupied 85% of the total activity in the PM fraction. PM H^+^-ATPase activity was measured on a U-2910 spectrophotometer (HITACHI, Tokyp, Japan) with wavelength A700.

### 4.7. Yeast Two-Hybrid Analysis

The coding sequence of *AHA2* (2607–2942 bp) and *PEPR2* (2282–3218 bp) fragments were cloned and cut with EcoR I and BamH I restriction enzymes, and the fragments were then linked into pGBKT7 and the pGADT7 vectors, respectively. Next, the yeast strain AH109 stored at −80 °C was taken out and activated in Yeast Peptone Dextrose Adenine (YPDA) solid medium, at 30 °C. Then, a single colony of activated AH109 was picked and cultured in 30 mL liquid YPDA medium until the OD_600_ was 2.0, at 30 °C, 250 rpm. Last, the fusion pGBKT7-PEPR2 and pGADT7-AHA2 constructs were co-transformed into the yeast strain AH109 using the lithium acetate transformation method [51]. We pipetted 100 uL of transformants onto the synthetic dropout (SD) medium (-Leu/-Trp) and incubated at 30 °C for 3 days. Then, we picked the transformants from synthetic dropout (SD) medium (-Leu/-Trp) and diluted them at different dilutions (10-1, 10-2, and 10-3) and incubated them in synthetic dropout (SD) medium (-Trp/-Leu/-His/-Ade) at 30 °C for 4 days. The primers used are listed in Supplementary Table S1.

### 4.8. BiFC Assay

For the Bimolecular fluorescence complementation (BiFC) assay, full-length CDS of PEPR2 and AHA2 amplified from the cDNA of wild-type seedlings were fused in-frame to the N-terminus and C-terminus of YFP to form PEPR2-nYFP and AHA2-cYFP, respectively. All of the constructs were transformed into *A. tumefaciens* strain GV3101 and then infiltrated into *Nicotiana benthamiana* leaves as described earlier [52]. Briefly, the Agrobacterium strains containing PEPR2-nYFP or AHA2-cYFP construct and p19 silencing plasmids were respectively cultured in 30 mL Luria-Bertani (LB) liquid medium until the OD_600_ reached 1.0. Then, we mixed the contained PEPR2-nYFP and AHA2-cYFP Agrobacterium strains with the p19 strain in an equal volume ratio. Last, the mixed strains were co-infected to the *Nicotiana benthamiana* leaves and the infected leaves were checked under a confocal microscope (LSM-710, Zeiss, Oberkochen, Germany), operated with excitation wavelength of 488 nm after injection for 2–3 days. The primers used are listed in Supplementary Table S1.

### 4.9. Statistical Analysis

For all experiments, three independent repetitions were performed. One-way analysis of variance (ANOVA) Tukey’s test was used for statistical analysis. Asterisks in the figures denote significant differences as follows: * *p* < 0.05, ** *p* < 0.01, and *** *p* < 0.001.

## Supplementary Materials

The following supplementary materials can be found at https://www.mdpi.com/1422-0067/21/21/7963/s1. Figure S1: Pep1 inhibits the root growth independent of K^+^, Na^+^, and Cs^+^ cations. Figure S2: The identification of *aha1–7*, *aha2–4,* and *aha7* mutants. Figure S3: AHA2 does not function redundant with AHA7 to regulate the Pep1 signaling in roots. Figure S4: The identification of aha2 pepr1 pepr2 triple mutant. Figure S5: PEPR2 primarily perceives the Pep1 signaling in roots. Table S1: Primers used in this study.
