# Supplementary material for: Danger-Associated Peptide Regulates Root Growth by Promoting Protons Extrusion in an AHA2-Dependent Manner in Arabidopsis
Source: Int J Mol Sci. 2020 Oct 27;21(21):7963. doi: 10.3390/ijms21217963 (PMC7663391; doi:10.3390/ijms21217963)
Supplement: Supplementary file 1 [file ijms-21-07963-s001.pdf]

Supplementary data

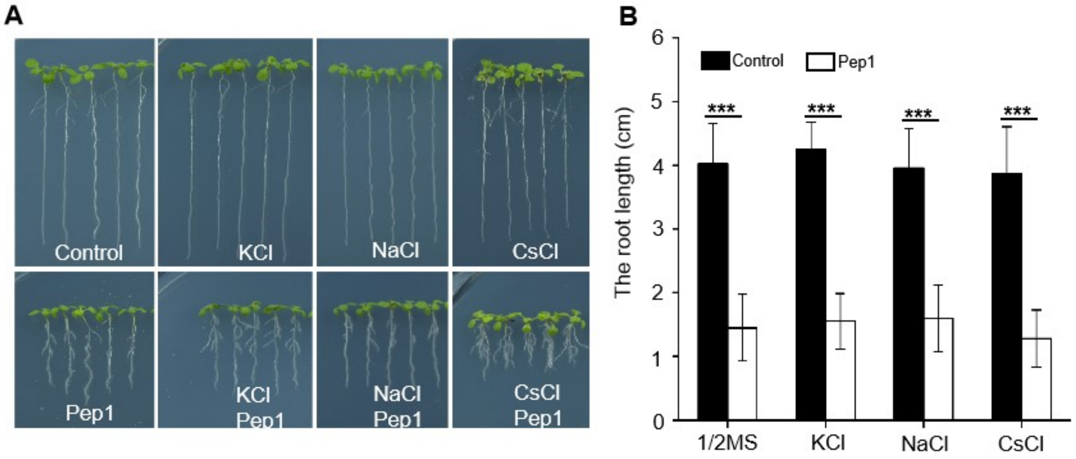

Supplementary figure 1. Pep1 inhibits the root growth independent on K^+, Na^+ and Cs^+ cations.

(A) The growth phenotype of wild type (WT) root. Three-day-old seedings were transferred onto half-strength MS medium supplemented with or without (Control) 50nM Pep1 in the presence or in the absence of 30mM KCl, 50mM NaCl or 2mM CsCl for 6 days. **(B)** The statistical analysis of the primary root length as indicated in **(A)**. Data are means ± SD from three replicate experiments (n=24 plants per treatment).

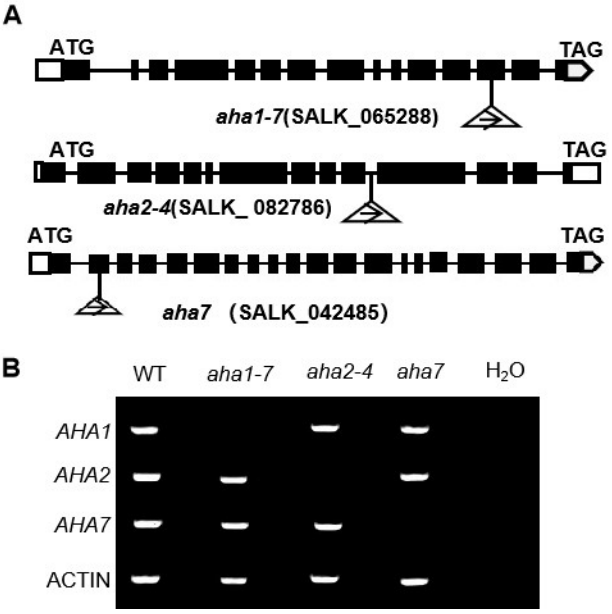

Supplementary figure 2. The identification of *aha1-7*, *aha2-4* and *aha7* mutants.

(A) Schematic map of T-DNA insertion locations of *aha1-7*, *aha2-4*, *aha7* mutants. Black boxes, lines and triangles represented exons, introns, and the position of the T-DNA insertion, respectively. The 5'- and 3'-UTRs are indicated by open boxes. ATG, translation start codon; TGA, stop codon. **(B)** RT-PCR detection of the transcriptional level of *AHA1*, *AHA2* and *AHA7* expression in wild type, *aha1-7*, *aha2-4*, and *aha7* mutants. Water was used as a negative control.

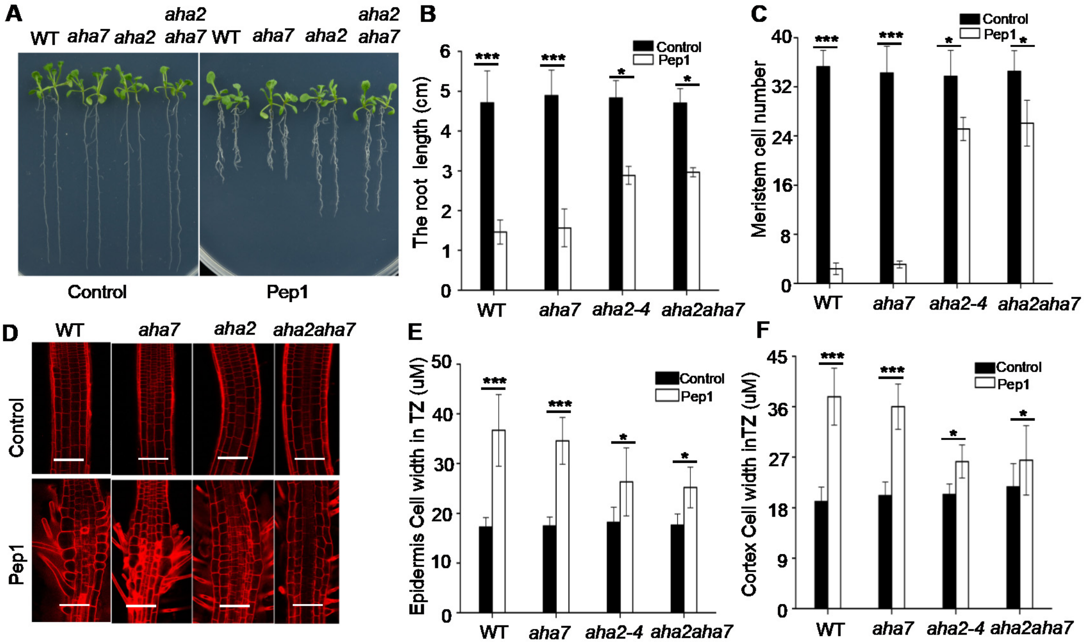

Supplementary figure 3. AHA2 does not function redundant with AHA7 to regulate the Pep1 signaling in root.

(A) The growth phenotype of wild type (WT), *aha7*, *aha2* and *aha2 aha7* roots. Three-day-old seedings were transferred on half-strength MS agar medium supplemented with or without (Control) 50 nM Pep1 for 6 days. **(B)** The statistical analysis of the primary root length in WT, *aha7*, *aha2* and *aha2 aha7* plants as indicated (A). Data are means±SD from three independent experiments, (n=15). **(C)** Quantitative analysis of numbers of meristematic cortex cells as indicated in **(A).** Data are means ± SD from three replicate experiments (n=24). **(D)** Longitudinal structures of roots in WT, *aha7*, *aha2* and *aha2 aha7* plants. 5-day-old seedings were transferred on half-strength MS agar medium supplemented with or without (Control) 100nM Pep1 for 24h, the roots were stained with 5 uM PI for 15s and photographed

under a confocal laser-scanning microscope. Similar results were obtained in three independent replicates. Bars=100um. **(E)** and **(F)** Quantitative analysis of epidermal and cortex cell width in TZ as indicated in **(D)**. Data are means±SD from three independent experiments (n = 32 cells from 8 roots per treatment). Asterisks in **(B)**, **(C)**, **(E)** and **(F)** indicate statistically significant differences (Tukey's test; *, P<0.05, **, P<0.01,***, P<0.001).

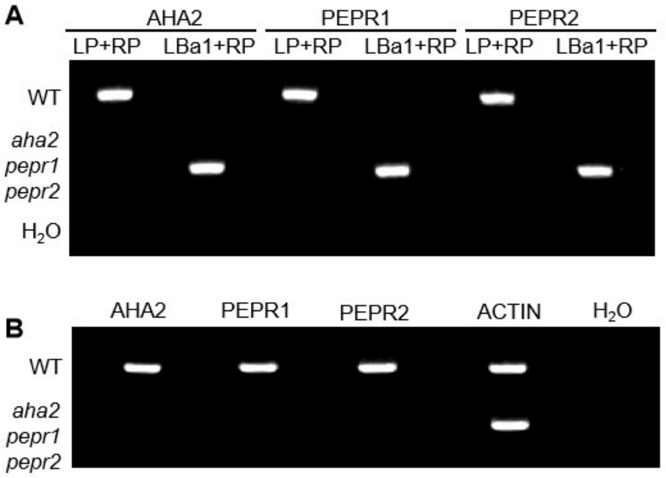

Supplementary figure 4. The identification of *aha2 pepr1 pepr2* triple mutant.

(A) Confirmation of the T-DNA insertion in the *aha2 pepr1 perp2* triple mutant by PCR. **(B)** RT-PCR detection of *AHA1*, *AHA2* and *AHA7* expression in wild type and *aha2 pepr1 pepr2* triple mutant. Water was used as a negative control.

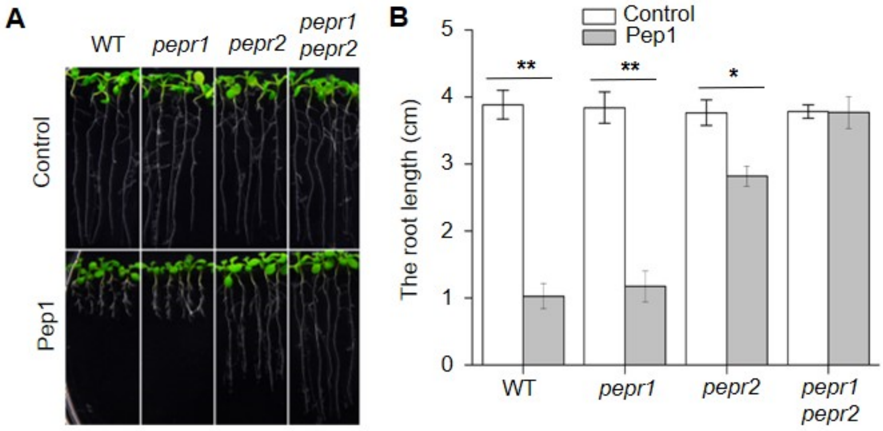

Supplementary figure 5. PEPR2 primarily perceives the Pep1 signaling in roots.

(A) The growth phenotype of wild type (WT), pepr1, pepr2 and pepr1 pepr2 roots.

Three-day-old seedings were transferred on half-strength MS agar medium supplemented with or without (Control) 50 nM Pep1 for 6 days. **(B)** The statistical analysis of the primary root length in WT, pepr1, pepr2 and pepr1 pepr2 plants as indicated **(A)**. Data are means±SD from three independent experiments, (n=15). Asterisks indicate statistically significant differences (Tukey's test; *, P<0.05, **, P<0.01).

Supplementary Table 1 Primers used in this study

Primer name	Primer sequences (5'-3')
(1) qRT-PCR analyze	
Actin2-F	CTGTTCTCTCCTTGTACGCCAGT
Actin2-R	CGGGTAATTCATAGTTCTTCTCGAT
AHA1-F	TTGAAGTTTGCCATTCGGTA
AHA1-R	GCCCATTGAGCTTCTCTTTC
AHA2-F	TTGTTGAACGTCCTGGAGCA
AHA2-R	AATTCCCAGTTGGCGTAAACC
AHA7-F	AGAAATAGCGCAACGGAACT
AHA7-R	TTGCAGCTGATTCAACCTTC
**(2) BiFC analysis**	
AHA2-BiFC-F	GACTAGTATGTCGAGTCTCGAAGATATCAAG
AHA2-BiFC-R	CGGGATCCCACAGTGTAG TGACTGGGAGT
PEPR2-BiFC-F	GACTAGTATGAGGAATC TTGGGTTACT CGA
PEPR2-BiFC-R	CCCCCGGGTGAACTGAA CCCGAAGTGC
**(3) Yeast two-hybrid analysis**	
PEPR2-F	GGAATTCCCGGTGCAAAAGAGAACCAAC
PEPR2-R	CGGGATCCAAATCTTTCACCACATCTCTCATC
AHA2-F	GGAATTCTTCAAGTTTGCCATTCGATACAT
AHA2-R	CGGGATCCCACATGTAGTGACTGGGAGTTTCA
**(4) Identification of *aha1-7, aha2-4, aha7* mutant**	
LBa1	TGGTTCACGTAGTGGGCCATCG
aha1-7-LP	ATGTCAGGTC TCGAAGATAT
aha1-7-RP	CTACACAGTG TAGTGATGTC
aha2-4-LP	ACCTCTGGCTCAAAATTGTCC
aha2-4-RP	CTCCAGGACGTTCAACAAAAG
aha7-LP	ATGACGGACATAGAAGCTCTC
aha7-RP	TTCGCTTTAG GGGCTAACTG
pepr1-LP	ACATCAGACGGACGTAAAACG
pepr1-RP	TGCAATTAGGTGATCCGAAAC
pepr2-LP	TCCAATGTGAGGCTCCATATC
pepr2-RP	TTCTCAAAACAAACTCACGGG
